# TNF-α differentially modulates subunit levels of respiratory electron transport complexes of ER/PR +ve/−ve breast cancer cells to regulate mitochondrial complex activity and tumorigenic potential

**DOI:** 10.1186/s40170-021-00254-9

**Published:** 2021-04-29

**Authors:** Anjali Shinde, Hyeryeon Jung, Hayun Lee, Kritarth Singh, Milton Roy, Dhruv Gohel, Han Byeol Kim, Minal Mane, Hitesh Vasiyani, Fatema Currim, Yu Ri Seo, Seojin Yang, Ara Cho, Eugene C. Yi, Rajesh Singh

**Affiliations:** 1 Department of Bio-Chemistry, Faculty of Science, The Maharaja Sayajirao University of Baroda, Sayajigunj, Vadodara, Gujarat 390002 India; 2 Department of Molecular Medicine and Biopharmaceutical Sciences, Graduate School of Convergence Science and Technology, Seoul National University, Seoul, 03080 South Korea; 3 Department of Cell and Developmental Biology, University College London, Gower Street, London, WC1E 6BT UK

**Keywords:** Breast cancer heterogeneity, TNF-α, Mitochondria, Metabolism, Inflammation

## Abstract

**Background:**

Tumor necrosis factor-α (TNF-α) is an immunostimulatory cytokine that is consistently high in the breast tumor microenvironment (TME); however, its differential role in mitochondrial functions and cell survival in ER/PR +ve and ER/PR −ve breast cancer cells is not well understood.

**Methods:**

In the current study, we investigated TNF-α modulated mitochondrial proteome using high-resolution mass spectrometry and identified the differentially expressed proteins in two different breast cancer cell lines, ER/PR positive cell line; luminal, MCF-7 and ER/PR negative cell line; basal-like, MDA-MB-231 and explored its implication in regulating the tumorigenic potential of breast cancer cells. We also compared the activity of mitochondrial complexes, ATP, and ROS levels between MCF-7 and MDA-MB-231 in the presence of TNF-α. We used Tumor Immune Estimation Resource (TIMER) webserver to analyze the correlation between TNF-α and mitochondrial proteins in basal and luminal breast cancer patients. Kaplan-Meier method was used to analyze the correlation between mitochondrial protein expression and survival of breast cancer patients.

**Results:**

The proteome analysis revealed that TNF-α differentially altered the level of critical proteins of mitochondrial respiratory chain complexes both in MCF-7 and MDA-MB-231, which correlated with differential assembly and activity of mitochondrial ETC complexes. The inhibition of the glycolytic pathway in the presence of TNF-α showed that glycolysis is indispensable for the proliferation and clonogenic ability of MDA-MB-231 cells (ER/PR −ve) as compared to MCF-7 cells (ER/PR +ve). The TIMER database showed a negative correlation between the expressions of TNF-α and key regulators of mitochondrial OXPHOS complexes in basal breast vs lobular carcinoma. Conversely, patient survival analysis showed an improved relapse-free survival with increased expression of identified proteins of ETC complexes and survival of the breast cancer patients.

**Conclusion:**

The evidence presented in our study convincingly demonstrates that TNF-α regulates the survival and proliferation of aggressive tumor cells by modulating the levels of critical assembly factors and subunits involved in mitochondrial respiratory chain supercomplexes organization and function. This favors the rewiring of mitochondrial metabolism towards anaplerosis to support the survival and proliferation of breast cancer cells. Collectively, the results strongly suggest that TNF-α differentially regulates metabolic adaptation in ER/PR +ve (MCF-7) and ER/PR −ve (MDA-MB-231) cells by modulating the mitochondrial supercomplex assembly and activity.

**Supplementary Information:**

The online version contains supplementary material available at 10.1186/s40170-021-00254-9.

## Introduction

Breast cancer is still a leading cause of death in women worldwide [1], hence it requires a further understanding of metabolic adaptations of different breast cancer subtypes, for the identification of alternative or novel drug targets. Broadly, breast cancer has been categorized as hormone-responsive ER/PR +ve representing an early benign tumor condition, whereas ER/PR −ve as aggressive and metastasis at a late stage. The tumor microenvironment (TME) of a solid tumor is complex and constitutes many different cell types including the recruitment of circulating monocytes and its differentiation to tumor-associated macrophage (TAM) [2]. The interaction of TAMs and breast cancer cells lead to an inflammatory milieu in TME which reprograms the genetic expression landscape of tumor cells leading to immune evasion and tumor progression. The mechanisms regulating these processes are emerging, however not well understood.

Inflammation in TME enhances tumor growth and metastasis in cancers of different origin like pancreatic, lung, and gastric [3–6] including breast cancer. Inflammation affects all phases of malignancy, including proliferation at the early stage, angiogenesis, progression, and tumor metastasis [7, 8]. The increased levels of several pro-inflammatory cytokines like TNF-α, IL-8, IL-10, and growth factors like TGF-β have been observed in the tumor microenvironment [8–10]. Despite the close association between inflammation and tumorigenesis, the mechanisms underlying the cytokine-mediated metabolic adaption and its impact on regulating the tumorigenic potential of breast cancer cells is not well understood.

TNF-α is a pleiotropic cytokine and acts as pro- or anti-tumorigenic depending on the type and stage of specific cancer. TNF-α level is high in tumors of different origin including breast cancer. Moreover, studies in the last decade had shown that mitochondria are emerging as a platform for assembly of the complexes regulating the NF-κB and IFN pathways, hence innate immunity during viral infections. Previously, we have reported that adaptor proteins like STING and NLRX1 localize to mitochondria and its contact site, which beyond their role in innate immunity, can act as tumor suppressor and modulate mitochondrial functions in the presence of TNF-α [11, 12]. This evidences suggest that mitochondria act as a signaling hub for integrating inflammatory and metabolic cues; however, their role in maintaining cancer cell metabolism and regulating the overall tumor growth within the altered cytokine milieu of a TME, needs to be further investigated. In the current study, we systematically investigated the TNF-α modulated mitochondrial proteome by employing quantitative mass spectrometry. We observed that TNF-α differentially modulate the subunits of oxidative phosphorylation (OXPHOS) complexes and mitochondrial functions to regulate the clonogenic and migration abilities of the breast cancer cells.

## Materials and methods

### Cell Lines used and the reagents

MCF-7 and MDA-MB-231 cells were cultured in Dulbecco’s modified Eagle’s medium (DMEM, Life Technologies, Carlsbad, CA, USA) media were supplemented with 10% v/v heat-inactivated fetal bovine serum (Life Technologies) 1% penicillin, streptomycin, and neomycin (PSN) antibiotic mixture (Life Technologies). Human TNF-α (premium grade) was purchased from MiltenylBiotec GmbH, Germany.2-deoxy-glucose, NAC (N-Acetyl Cysteine) were purchased from Sigma-Aldrich, USA. CM-H_2_DCFDA and MitoSOX^TM^ Red were purchased from Molecular Probes Inc., USA.

### Mitochondria isolation and quality control

Cells were seeded at 3 × 10^6^ density and after overnight incubation cells were treated as indicated. The cells were collected and passed through 24-G × 1″ syringe 50–60 times using Sucrose-Tris mitochondria isolation buffer (0.25 M Sucrose, 10 mM Tris HCl, and 1X protease inhibitor). After centrifugation at 600×*g* for 10 min, the supernatant was collected and centrifuged at 8000×*g*. The obtained pellet (mitochondrial fraction) was washed thrice with the isolation buffer and lysed in RIPA lysis buffer. Purity of mitochondrial fraction was checked by western blotting using antibodies against Actin, Tom20, SDHA, and UQCRC2.

### Sample preparation and digestion

Isolated mitochondrial fractions were lysed in RIPA lysis buffer (Thermo Scientific, Rockford, IL, USA) with protease inhibitor (Roche Diagnostics, Mannheim, Germany) and phosphatase inhibitor cocktail (Roche Diagnostics), followed by a brief sonication on ice. The cells were sonicated and centrifuged for 15 min at 24,000×*g* at 4 °C and the supernatant was transferred to a new tube. Protein concentration was determined using BCA assay kit (Thermo Scientific). Protein samples were fractionated on 4–12% Bis-Tris Gels (Invitrogen, Carlsbad, CA, USA) and stained with Coomassie Brilliant Blue (Sigma-Aldrich, St. Louis, MO, USA). Each gel lane was cut into ten pieces and subjected to in-gel tryptic digestion following the general protocol [13]. Briefly, protein bands were excised, destained, washed, and further reduced with 20 mM DTT and alkylated with 55 mM iodoacetamide. After dehydration, the proteins were digested with 13 ng/ml sequencing-grade modified porcine trypsin (Promega, Madison, WI, USA) in 50 mM ammonium bicarbonate overnight at 37 °C. Peptides were extracted from the gel slices in 50% (v/v) ACN and 5% (v/v) formic acid and dried under vacuum.

### Mass spectrometry analysis

Peptides were resuspended in 25 μL Solvent A (0.1% formic acid in water, pH 2.0) and 5 μL sample was loaded onto an analytic column (PepMap, 75 μm ID × 50 cm 3 μm, ES803, Thermo Fisher Scientific, San Jose, CA, USA) interfaced with a nano-ultra-HPLC system (EasynLC, Thermo Fisher Scientific) and separated with a linear gradient of 5–32% Solvent B (0.1% formic acid in ACN), time (B%) 0∼12 min (5% solvent B), 97 (40%), 105 (70%), 117 (70%), and 120 (2%), for 120 min at a flow rate 300 nL/min. MS spectra were recorded on a Q-Exactive Orbitrap mass spectrometer (Thermo Fisher Scientific). The standard mass spectrometric condition of the spray voltage was set to 2.2 kV and the temperature of the heated capillary was set to 250 °C. The full MS scans were acquired in the mass analyzer at 400–1400 m/z with a resolution of 70,000 and the MS/MS scans were obtained with a resolution of 17,500 by normalized collision energy of 27 eV for high-energy collisional dissociation fragmentation. The automatic gain control target was 1 × 10^5^, the maximum injection time was 120 ms, and the isolation window was set to 2.0 *m/z*. The Q-Exactive was operated in a data-dependent mode with one survey MS scan followed by ten MS/MS scans, and the duration time of dynamic exclusion was 20 s.

### Database search

Collected MS/MS data were searched against the decoy UniProt human database (version 3.83, 186 578 entries) by Proteome Discoverer 2.2 (PD 2.2, Thermo Scientific) software. Precursor and fragment ion tolerance were set to 10 ppm and 0.5 Da, respectively. Trypsin was chosen as the enzyme with a maximum allowance of up to two missed cleavages. Carbamidomethyl (+ 57.02) of cysteine was considered as the fixed modification, while the variable modification was set for methionine oxidation (+ 15.99). The result filtration parameters of PD 2.2 were set as follows: peptide and protein identifications were accepted if they could be established at greater than 95% and 99% probability, respectively, as specified by the Peptide and Protein Prophet algorithm and if the protein identification contained at least two identified peptides with a false discovery rate ≤ 0.1%.

### Relative protein quantification and bioinformatics analysis

Relative protein quantitation was accomplished using spectral counting. The MS/MS data were normalized to compare the abundances of proteins between samples using PD 2.2 software. The normalized spectral counts from triplicate analyses of the MCF-7 and MDA-MB-231 cells treated or untreated with TNF-α were compared using the R program [14] with power-law global error model (PLGEM; version 1.50.0) software used to determine signal-to-noise ratio and *P*-value [15, 16]. We filtered statistically significant differentially expressed proteins (DEPs) using 0.01 as a p-value threshold. Then we refined spectral count readouts for the proteins within the range of 0.01≤ p-value ≤0.05 using the Moment Adjusted Imputation (MAI) equation [17] to identify DEPs with statistical significance with more sensitivity. After the MAI refinements, we have determined the p-value with PLGEM and filtered statistically significant DEPs using 0.01 as a p-value threshold. The subcellular localization and functional annotation of the identified proteins were classified using Ingenuity Pathway Analysis (IPA, QIAGEN Inc., Valencia, CA, US) and Protein Analysis through Evolutionary Relationships Classification System (PANTHER, version 7.2,) [18].

Kaplan-Meier survival analysis was used to estimate the association of the gene’s expression with survival of patients.

### BN-PAGE

MCF7 and MDA-MB231 cells were seeded at density 3 × 10^6^/100 mm dish. After overnight incubation cells were treated as required. Mitochondria from MCF-7 and MDA-MB231 cells were isolated in Tris-Sucrose buffer as described above and 50 μg pellets were solubilized as per manufacturer’s protocol (Thermo Fisher Scientific) and BN-PAGE was performed on Native PAGE Novex3%–12% Bis-Tris Protein Gels (ThermoFisher Scientific). In-gel enzyme activity of different OXPHOS complexes was analyzed on gradient Bis-Tris gel.

Substrate: for complex I, 1 mg NADH and 25 mg NTB was  prepared in 2 mM Tris-HCl (pH 7.4), and for complex IV, 5 mg DAB and 10 mg cytochrome C in 50 mM potassium phosphate buffer (pH 7.4) was used for in-gel activity. For complex III and complex IV combined, 10 mg 3,3′ diaminobenzidine tetrachloride (DAB) and 25 mg cytochrome C in 25 ml of 50 mM sodium phosphate buffer (pH 7.2) was used.

### Spectrophotometric analysis of mitochondrial complex I and complex II assays

MCF-7 and MDA-MB-231 cells were seeded at the density of 5 × 10^5^ cells/well in the 6-well plate. The cells were treated as indicated, harvested, and washed with cold DPBS. The cells were subjected to 2–3 freeze-thaw cycles in a freeze-thaw complete solution (0.25 M sucrose, 20 mM Tris-HCl (pH 7.4), 40 mM KCl, 2 mM EDTA supplemented with 1 mg/ml fatty acid-free BSA, 0.01% Digitonin and 10% Percoll). The cells were washed again with the freeze-thaw solution devoid of digitonin and resuspended in complex I assay buffer (35 mM potassium phosphate (pH 7.4), 1 mM EDTA. 2.5 mM NaN_3_,1 mg/ml BSA, 2 μg/ml antimycin A, 5 mM NADH). Complex I activity was measured by monitoring the decrease in absorbance at 340 nm after the addition of 2.5 mM acceptor decylubiquinone indicating the oxidation of NADH.

Similarly, for complex II activity, cells were seeded at a density of 1.5 × 10^6^/60 mm dish. The cells were harvested and washed with cold DPBS. The cells were suspended in 0.5 ml of 20 mM hypotonic potassium phosphate buffer (pH 7.5) and lysed using a 24-G sterile syringe and subjected to freeze-thaw cycle. The cell lysate (80 μg) was added to the 1 ml of complex II assay buffer (0.1 M potassium phosphate (pH 7.5), 50 mg/ml BSA, 100 mM NaN_3_, 200 mM succinate) and incubated at 37 °C. Complex II activity was measured for 6 min by monitoring the decrease in absorbance at 600 nm after the addition of 2.5 mM acceptor decylubiquinone and DCPIP.

### ATP assay

MCF-7 and MDA-MB-231 cells were seeded in a density of 5 × 10^4^ in 24 well plates. ATP levels were measured in control and treatment conditions by an ATP dependent luciferase assay using an ATP determination kit (Molecular Probes/Life Technologies, ON, Canada).

### Assay of intracellular and mitochondrial ROS

ROS levels and mitochondrial ROS were measured by CM-H_2_DCFDA (10 μM) and MitoSOX^TM^ Red (5 μM) staining, respectively. Briefly, MCF-7 and MDA-MB-231 cells were plated at the density of 1.5 × 10^5^ cells/well in 24-well plates. The cells were treated and stained with indicated reagent and monitored under a fluorescence microscope (Olympus IX81 microscope; Olympus, Tokyo, Japan). A minimum of 5 images and 80–100 cells were used for analysis.

ROS levels were also quantified by fluorometry. Briefly, MCF-7 and MDA-MB231 cells were treated and stained with CM-H_2_DCFDA (12.5 μM) in DPBS for intracellular ROS quantification and MitoSOX Red (2.5 μM) in DMEM for mitochondrial ROS quantification. The cells were washed with DPBS and normalized to1 × 10^6^ cells/ml. Fluorescence intensity was quantified by a fluorometer (Hitachi High-Technologies Corp., Japan) with excitation/emission at 495/520–540 nm and 510/570–600 nm, respectively.

### MTT assay

MCF-7 and MDA-MB-231 cells were seeded (5000 cells/well) in 96-well plate. The cells were treated as indicated and cell viability was determined using the standard MTT [3-(4,5-dimethylthiazol-2-yl)-2,5-diphenyltetrazoliumbromideassay. The purple formazan crystals were dissolved in DMSO, transferred in a 96-well plate (100 μL/well) and the absorbance was recorded on a microplate reader at a wavelength of 570 nm.

### Clonogenic assay

MCF-7 and MDA-MB-231 cells (2000 cells/well) were seeded in 6 well plates and treated as per requirements. Cells were incubated till single clones were visible and later were fixed using methanol and stained using 0.2% crystal violet as described earlier [19].

### Survival analysis

BRCA patients in The Cancer Genome Atlas (TCGA) database were ranked by the level of NDUFB1, SDHA, and COX7C expression and divided into two groups: top quarter and low quarter in expression level. These groups were analyzed in Kaplan-Meier survival plot to estimate the correlation between the gene’s expression level and survival of patients using OncoLnc [20].

### Scratch assay

Scratch assay was performed in MCF-7 and MDA-MB-231 cells. Cells were seeded at a density of 2.5 × 10^5^ cells per well in 12 well plates. After overnight incubation cells were treated and a vertical wound was created using a sterile P200 micropipette tip. At zero time point, images of each scratch were taken using Nikon Ti-2 eclipse inverted fluorescent microscope at × 10 and were analyzed after 24 h of treatment. Migration rate was measured using ImageJ software which measures open area at a different time interval. The percentage of open area in each condition was plotted.

### Statistical analysis

Data are expressed as mean ± SEM of two or three independent experiments. Unpaired two-tailed Student’s *t*-test was performed. GraphPad Prism was used to perform all statistical analyses.

## Results

### TNF-α differentially modulates mitochondrial proteome in ER/PR +ve (MCF-7) and ER/PR −ve (MDA-MB-231) breast cancer cells

**Fig. 1:**
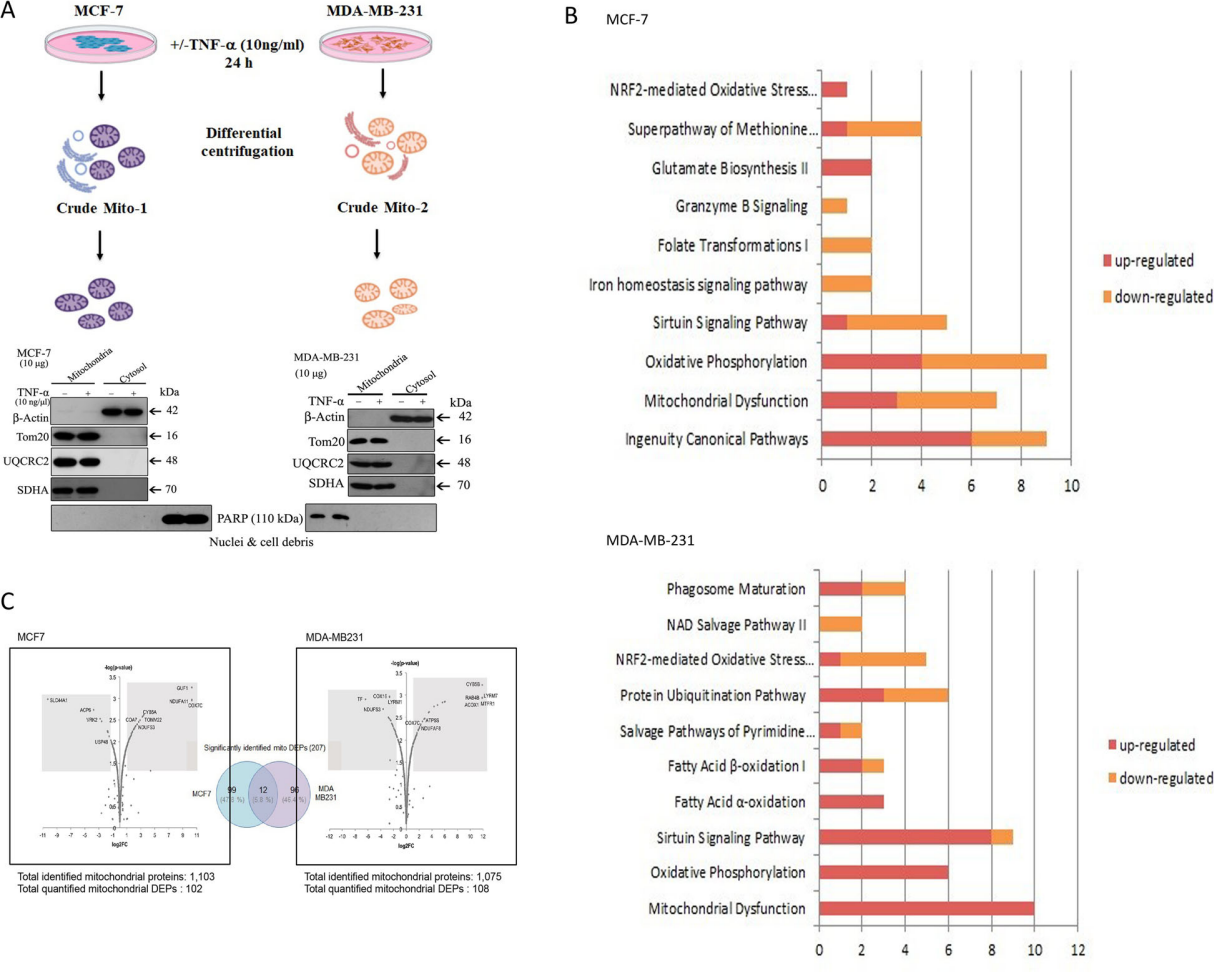
Quantitative proteomic analysis of TNF-α regulated mitochondrial proteins in breast cancer cells. **a** Immunoblot analysis of isolated mitochondria. **b** Molecular functions of DEPs by IPA tool. The *x*-axis indicates the number of proteins, red bar as upregulated, and orange bar as downregulated proteins. **c** Volcano plots of DEPs displayed in the *p*-value (−log10) versus signal-to-noise ratio (STN). The *p*-value and STN were determined by power law global error model (PLGEM) statistical analysis for label-free quantification

To identify TNF-α modulated differentially expressed mitochondrial proteins in MCF-7 (ER/PR +ve) and MDA-MB-231 (ER/PR −ve) cells, we performed quantitative proteomic analysis of mitochondrial proteins of both cell lines in the absence and presence of TNF-α. Mitochondrial fractions were prepared from MCF-7 and MDA-MB-231 cells and the purity was assessed by western blotting using selected mitochondrial marker proteins (Tom20, UQCRC2, and SDHA) including nuclei (PARP), cytosol (β-actin) (Fig. 1a). The mitochondrial fraction of both MCF-7 and MDA-MB-231 showed a high level of mitochondrial proteins of TOM20, UQCRC2, and SDHA, whereas PARP, a marker of nuclei, was not detected. LC-MS/MS data of MCF-7 +/- TNF-α and MDA-MB-231 +/- TNF-α were searched against the decoy UniProt human database and identified 1077 and 1150 mitochondrial proteins (peptide probability > 95%; protein probability > 99%) for MCF-7 and MDA-MB-231 cells, respectively. The list of identified mitochondrial proteins were further compared with mitochondrial protein databases; MitoCarta [21] and Gene Ontology [22]. It was observed that 57.5% of proteins overlapped between MDA-MB-231 and MCF-7 cells (Fig. 1c).

The quantitative statistical analysis by integrated PLGEM-STNMAI proteomics of triplicate LC-MS/MS data with the *p*-value threshold 0.01, identified 108 (62 upregulated and 49 downregulated proteins) and 111 (81 upregulated and 27 downregulated proteins) differentially expressed proteins (DEPs) in MCF-7 and MDA-MB-231, respectively (Fig. 1c), in the presence of TNF-α. Ingenuity pathway analysis (IPA) indicated that DEPs were involved in mitochondrial function (20%), Sirtuin Signaling Pathway (18%), oxidative phosphorylation (12%), protein ubiquitination (12%), and NRF2-mediated oxidative stress (10%) in MCF-7 cells. Interestingly, IPA analysis of DEPs in MDA-MB-231, ER/PR (−ve) cell line showed that proteins involved in mitochondrial dysfunction, oxidative phosphorylation, sirtuin signaling, and fatty acid β-oxidation were enriched in the presence of TNF-α as compared to MCF-7 cell lines (Fig. 1b).

The functional annotation of the DEPs of MCF-7 and MDA-MB-231 cell lines using IPA and hierarchical clustering analysis (Mev software) (Supplementary Figure 1A) showed a distinct cluster of genes regulating specific pathways were modulated in the presence of TNF-α. Cluster 1 shows DEPs between MCF-7 and MDA-MB-231 cells in the presence of TNF-α, associated with PPARα/RXRα activation, pyrimidine deoxyribonucleotides de novo biosynthesis, and salvage pathways of pyrimidine ribonucleotides. Cluster 2 shows that proteins downregulated in MCF-7 and upregulated in MDA-MB-231 cells in the presence of TNF-α, are associated with mitochondrial function, sirtuin signaling pathway, oxidative phosphorylation, oxidative ethanol degradation, fatty acid α-oxidation, TCA cycle, and glutamate biosynthesis. The results here suggest that the TNF-α differentially modulates mitochondrial proteome in both ER/PR +ve (MCF-7) and ER/PR −ve (MDA-MB-231) cells.

### TNF-α differentially regulates the level of critical proteins involved in mitochondrial ETC complex assembly

**Fig. 2:**
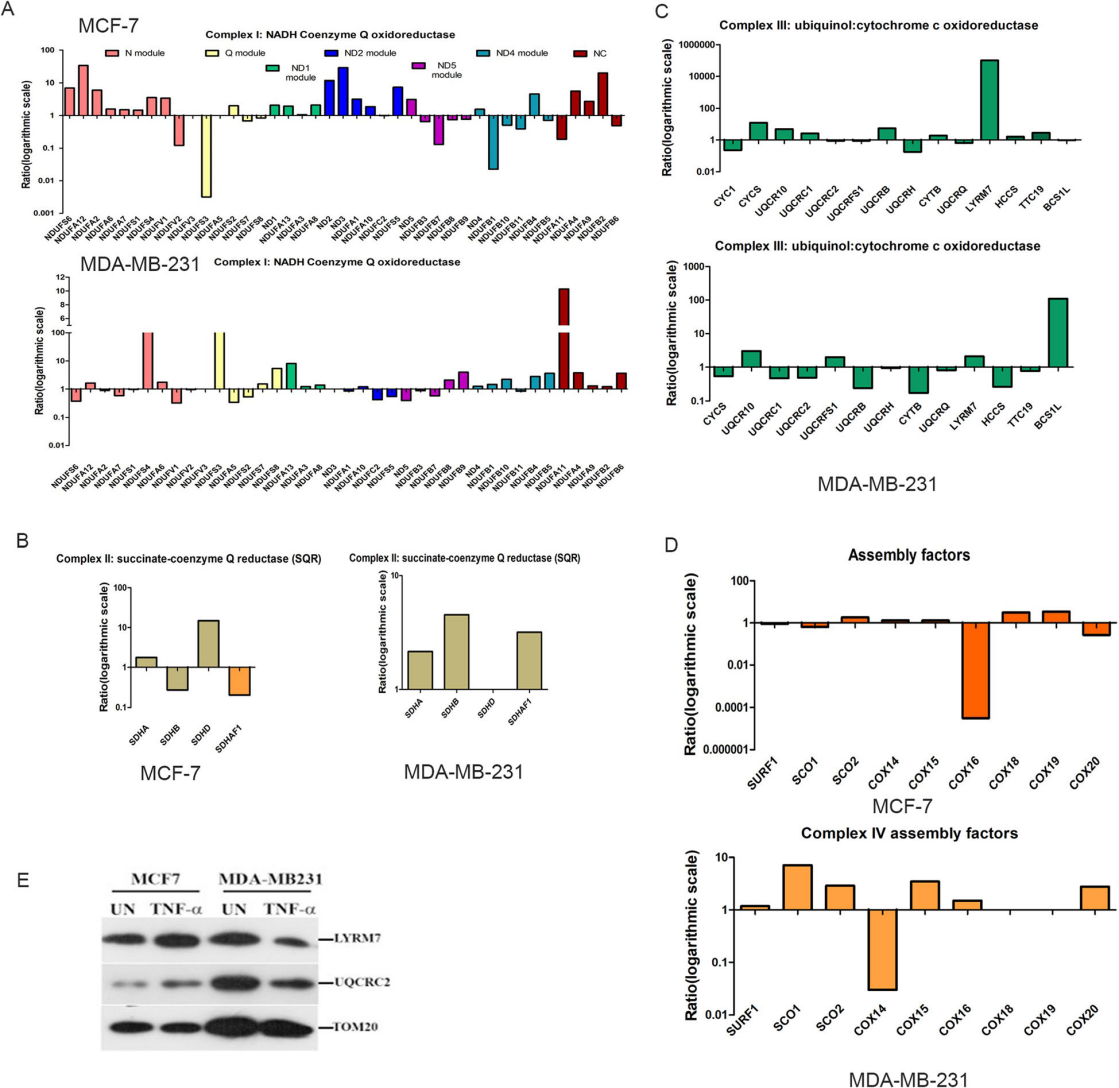
Differentially expressed proteins identified by proteomics correlates with TNF-α regulated mitochondrial function in breast cancer cells. The raw protein abundance count of mitochondrial protein subunits from untreated and TNF-α treated triplicates from both cells were exported and plotted as a ratio of log-fold change. The encircled proteins show the log-fold change of unique proteins in both cells. **a** The protein subunits of the CI complex are clustered and color-coded into respective modules according to their function. NC represents a non-characterized component of CI complex. **b**–**d** The log-fold change of unique proteins for CII, CIII, and CIV in both cell lines respectively (**e**) western blot analysis of complex III proteins UQCRC2 and LYRM7 in MCF-7 and MDA-MB-231 in the presence of TNF-α

The remodeling of electron transport chain complexes assembly and/or activity is important for the bioenergetic adaptation in cancer cells; hence, we focused specifically on the individual protein constituents of each respiratory complex. All the known subunits of electron transport chain (ETC) complexes including complex I, II, III, and IV were analyzed both in MCF-7 and MDA-MB-231 in the presence of TNF-α. The levels of NDUFS3 (a N module component) and NDUFB1 (the component of ND4 module) significantly decreased in MCF-7 cells in the presence of TNF-α and, however, increased in MDA-MB-231(Fig. 2a). The levels of NDUFA11, the matrix-facing subunit of CI increased several folds in MDA-MB-231 cells and decreased in MCF-7 in the presence of TNF-α. This suggests that the levels of mitochondrial complex I proteins are differentially altered in ER/PR +ve(MCF-7) and ER/PR −ve(MDA-MB-231) cells in the presence of TNF-α.

Complex II is the smallest mitochondrial complex and unique as it forms a part of the TCA cycle as well as a part of ETC hence directly linking metabolism and oxidative phosphorylation; we, therefore, analyzed the levels of complex II subunits in MCF-7 and MDA-MB-231 cells in the presence of TNF-α. MCF-7 showed increased levels of SDHD subunit whereas other subunits SDHA and the assembly factor, SDHAF1, decreased in the presence of TNF-α (Fig. 2b). MDA-MB-231 cells treated with TNF-α showed no change in SDHD level whereas the level of SDHA and SDHB increased in mitochondria. The level of SDHAF1 was significantly high in mitochondria of TNF-α treated MDA-MB-231 cells as compared to MCF-7.

Mitochondrial complex III is an important complex as it accepts electrons both from complex I and complex II via the acceptor, ubiquinone. The alteration of complex III may lead to oxidative stress and accumulation of oncometabolite leading to increased cell proliferation [23]. We further analyzed the level of complex III subunits from the mitochondrial proteome of both MCF-7 and MDA-MB-231 cells in the presence of TNF-α. Levels of most subunits of complex III remained same both in MCF-7 and MDA-MB-231 in the presence of TNF-α. Interestingly, LYRM7, a protein having the LYR (Leucine, Tyrosine, Arginine) consensus sequence binds to HSC20 and facilitate incorporation of Fe-S cluster into UQCRFS1 in complex III [24, 25] during assembly of the respiratory chain showed altered levels in both cell lines. The level of LYRM7 significantly increased in MCF-7 in the presence of TNF-α as compared to MDA-MB-231 (Fig. 2c). To confirm this, the expression levels of LYRM7 levels were also analyzed by western blotting. LYRM7 protein levels also increased in mitochondrial fraction of TNF-α treated MCF-7 cells whereas decreased in MDA-MB-231 cells (Fig. 2e). UQCRC2, a complex III subunit also decreased in TNF-α treated MDA-MB-231 mitochondrial fraction and correlated with proteomics data (Fig. 2e). Similarly, BCS1L, a 419-amino-acid chaperone protein, is a member of the family called AAA; is localized in the inner membrane of the mitochondria; and is presumed to facilitate the insertion of Rieske Fe/S protein into precursors to complex III [26]. We also analyzed the level BCS1L in mitochondrial proteome and observed increased levels in mitochondria of MDA-MB-231 cells in the presence of TNF-α whereas remained unchanged in MCF-7 cells.

Complex IV subunit levels were also analyzed in mitochondrial fraction of both MCF-7 and MDA-MB-231. We did not observe any significant changes in the levels of different cytochrome-c oxidase (COX) complex subunits. However, interestingly the assembly factors required for the complex IV biogenesis were differentially regulated in the presence of TNF-α in the breast cancer cells. COX14 assembly factor plays an important role in the translation of the COX1, the main constituent of complex IV [27]. Its abundance decreased significantly in the mitochondria of MDA-MB-231 as compared to MCF-7 in the presence of TNF-α (Fig. 2d). Similarly, another assembly factor, COX16, was downregulated in MCF-7 cells as compared to MDA-MB-231. Altogether, the above evidence from quantitative mitochondrial proteomics and immunoblotting suggests that protein levels of specific subunits of mitochondrial ETC complexes are differentially regulated (Supplementary figure 2) which may, in turn, modulate the assembly and/or activity in the presence of TNF-α.

### TNF-α differentially regulates mitochondrial supercomplex assembly and activity in ER/PR +ve (MCF-7) and ER/PR −ve (MDA-MB-231) breast cancer cells

**Fig. 3:**
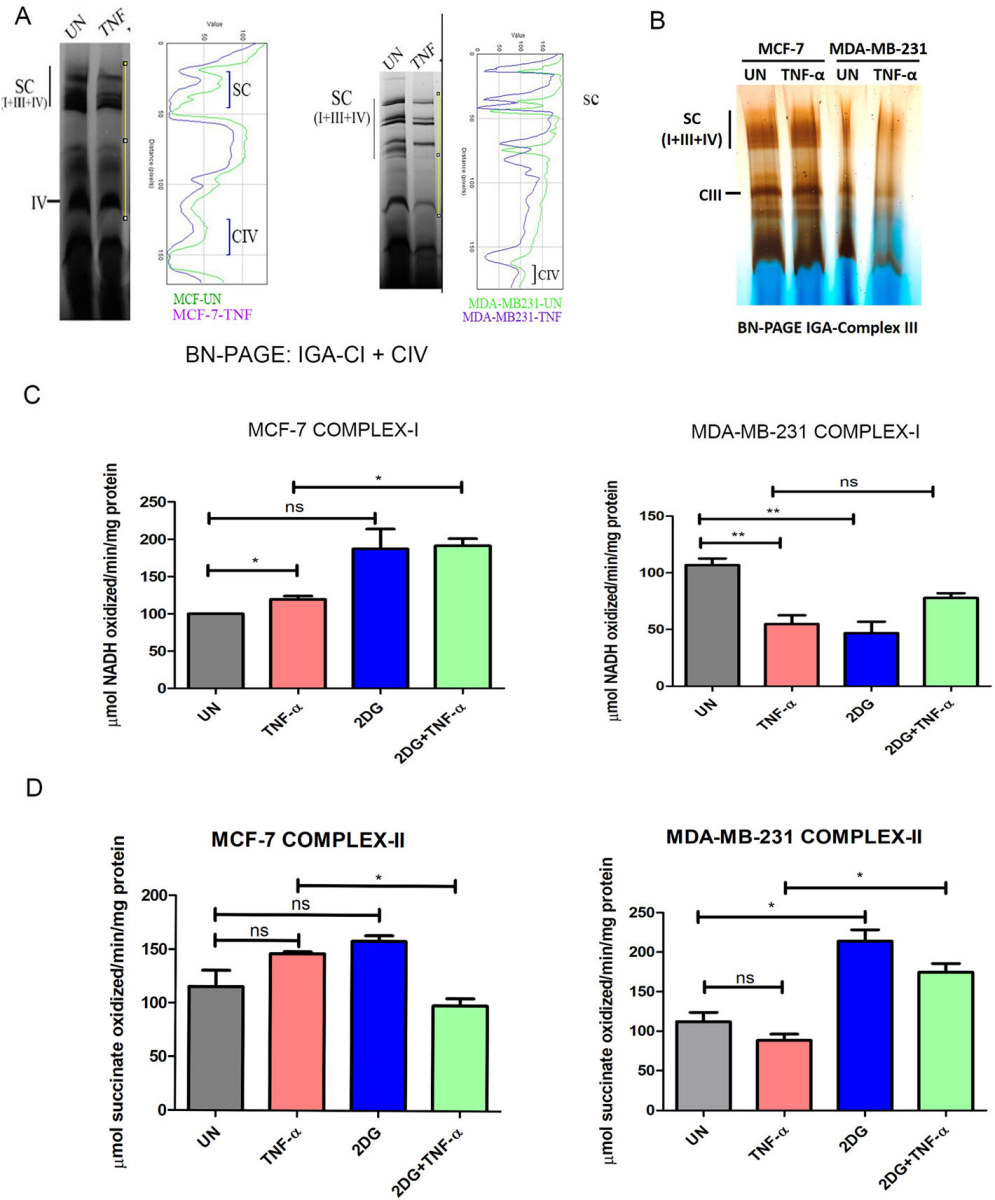
TNF-α alters the OXPHOS assembly and activity in breast cancer cells. **a** MCF-7 and MDA-MB-231 cells were treated with TNF-α (10 ng/μl) for 24 h. After treatment, the mitochondrial fraction was isolated and further analyzed by BN-PAGE, and in-gel enzyme staining for CI and CIV was performed. **b** Complex III in-gel activity was analyzed by BN-PAGE and enzyme staining. **c**, **d** MCF-7 and MDA-MB-231 cells were treated with TNF-α (10 ng/μl) and 2-DG (10 mM) either alone or in combination for 24 h and complex I and complex II activity was measured spectrophotometrically

To understand the implication of TNF-α modulated subunits of mitochondrial ETC complexes, we analyzed the organization and activity of ETC complexes from both MCF-7 and MDA-MB-231 cells in the presence/absence of TNF-α using Blue native-PAGE. We observed that TNF-α decreased the levels, as well as the activity of supercomplex (SC) containing complex I and complex IV in both the cell line; however, this decrease was significantly higher in MDA-MB-231 cells in the presence of TNF-α (Fig. 3a). A significant decrease in individual complex III activity was observed in MDA-MB-231 in the presence of TNF-α compared to MCF-7 (Fig. 3b).To further quantify the enzyme kinetics of individual ETC complexes, we monitored the specific activity of complex I and complex II using the spectrophotometric assay in MCF-7 and MDA-MB-231 cells in the presence of TNF-α either alone or combination with 2-deoxyglucose (2-DG). 2-DG, the glucose analog, inhibits glycolysis and decreases the growth of tumor cells, which are primarily dependent on the glycolytic pathway. Complex I activity significantly decreased in the presence of TNF-α in MDA-MB-231 as compared to MCF-7 cells. The inhibition of glycolysis with either 2DG alone or in combination with TNF-α increased complex I activity in MCF-7 cells. On the other hand, the activity of complex I decreased significantly upon inhibition of glycolysis by 2-DG in MDA-MB-231 but remained unchanged in the presence of both 2-DG and TNF-α (Fig. 3b). These results suggest that TNF-α negatively regulates complex I activity in MDA-MB-231 cells which depends on glycolysis to maintain its activity in contrast to MCF-7.

The measurement of complex II activity revealed no significant changes either in MCF-7 or MDA-MB-231 in the presence of TNF-α. However, treatment with 2-DG alone or in combination with TNF-α significantly increased complex II activity in MDA-MB-231 suggesting a compensatory response by complex II to maintain the overall ETC function during glycolytic inhibition. Altogether, these results indicate that TNF-α modulates the levels of critical components of complex I, complex III, and complex IV, hence differentially regulate the organization and activity of ETC complexes in MDA-MB-231 cells (ER/PR −ve) and MCF-7 cells (ER/PR +ve) in the presence of TNF-α.

### TNF-α downregulates ATP levels and enhances ROS generation in triple-negative MDA-MB-231 cells

**Fig. 4:**
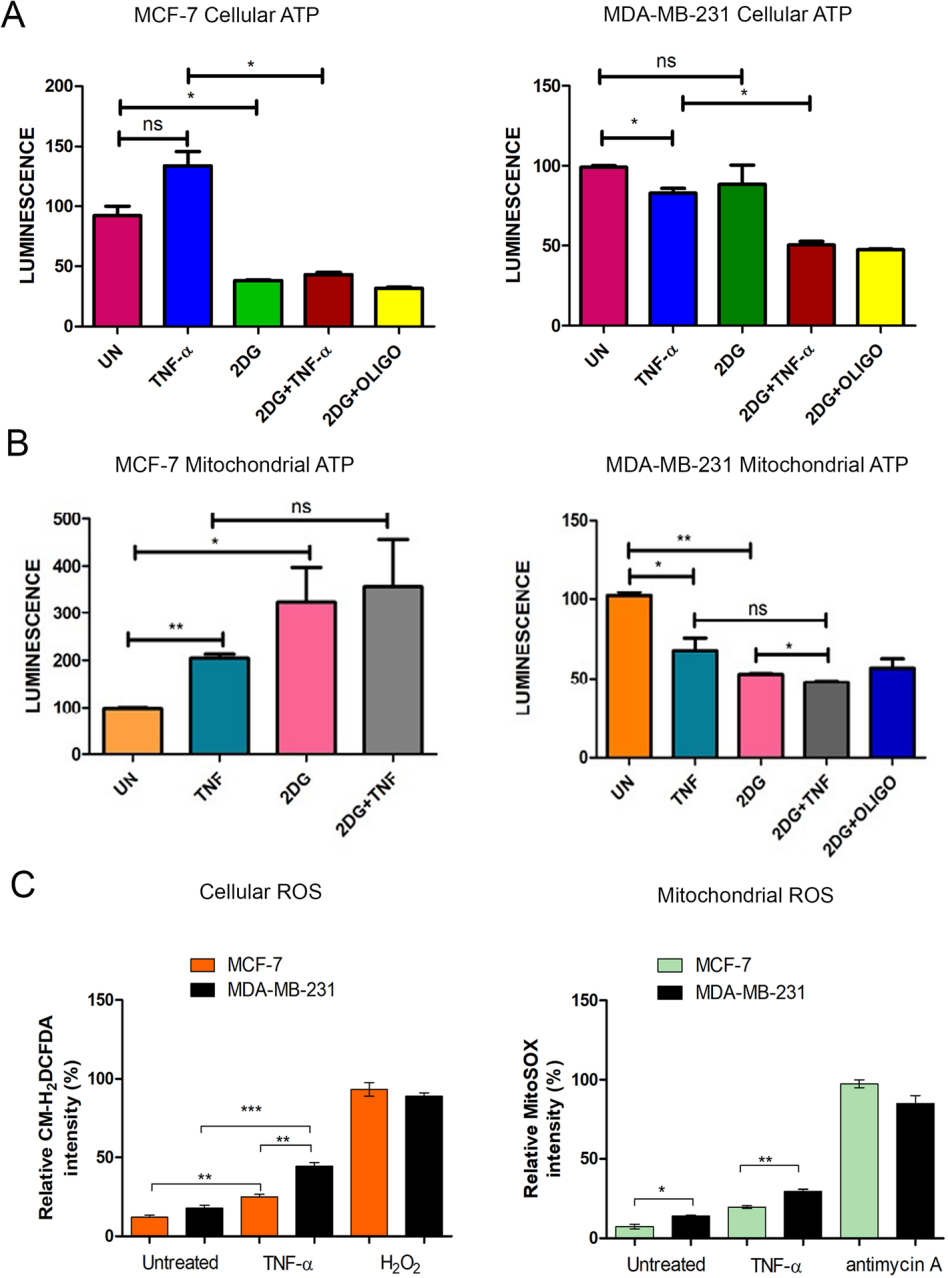
TNF-α decreases ATP levels and increases ROS generation in MDA-MB-231 cells. **a** MCF-7 and MDA-MB-231 cells were treated with TNF-α either alone or in combination with 2-DG (10 mM) and the total steady-state ATP levels were determined by the luciferase-based assay as described in methodology. **b** As in **a**, mitochondria from respective cells were isolated upon treatment and ATP levels were measured. Oligomycin (5 μg) treatment for 20 min was used as a positive control. **c** MCF-7 and MDA-MB-231 cells were treated with TNF-α. After treatment, the cells were stained with fluorogenic ROS-sensitive dye and relative fluorescence was monitored as described in methodology. H_2_O_2_ (100 μM) and antimycin A (10 μg) treatment for 2 h were used as a positive control

To investigate the effect of TNF-α regulated ETC complexes assembly/activity on the mitochondrial bioenergetic status of both the cells, we analyzed the level of ATP and ROS in the presence/absence of TNF-α. In accordance with the above results, TNF-α treatment significantly decreased both total cellular and mitochondrial ATP level in MDA-MB-231 cells but not in MCF-7 cells. MCF-7 cells cultured in the presence of 2-DG in the presence/absence of TNF-α showed a significant decrease in total ATP levels; however, the mitochondrial ATP levels increased significantly under all treatment conditions suggesting an upregulated ETC function (Fig. 4a, b). In contrast, MDA-MB-231 cells displayed increased sensitivity to a decrease in both mitochondrial and total cellular ATP levels in the presence of TNF-α with or without 2-DG (Fig. 4a, b). This result suggested that MDA-MB-231 cells are strongly dependent on the glucose-pyruvate axis for substrate oxidation and ATP generation by OXPHOS, which is altered in the presence of TNF-α. Similarly, both intracellular and mitochondrial ROS levels significantly increased in both cell lines however MDA-MB-231 cells displayed an enhanced ROS generation in the presence of TNF-α (Fig. 4c) as compared to MCF-7. Altogether, these results strongly suggest that TNF-α alters the mitochondrial bioenergetic status of MDA-MB-231 cells by negatively regulating the ETC complexes activity leading to a decrease in ATP levels and increased ROS generation.

### TNF-α modulated mitochondrial functions differentially regulate migration and clonogenic ability of breast cancer cells

**Fig. 5:**
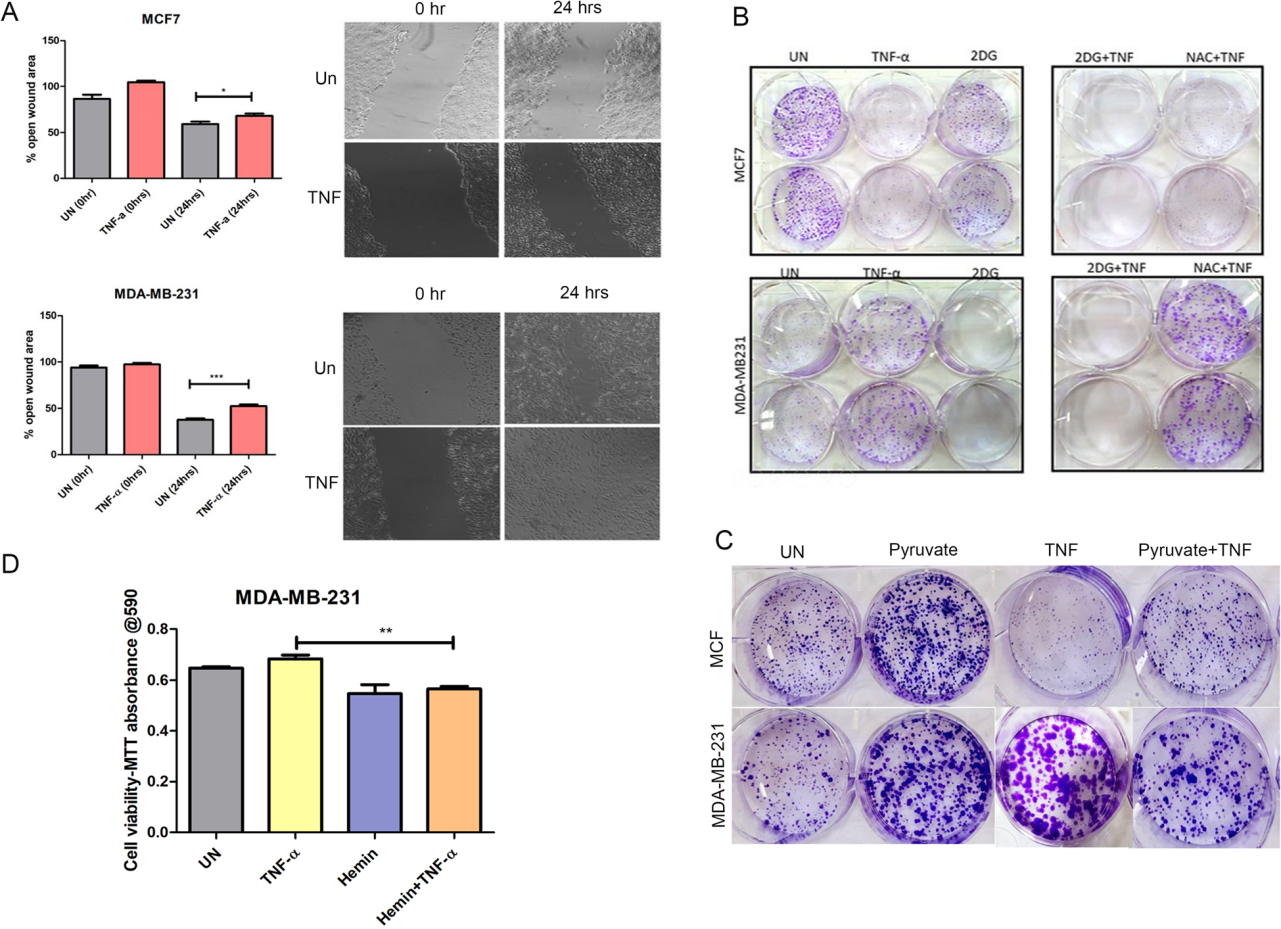
TNF-α sensitizes cell death and decreases growth in MDA-MB-231 cells. **a** MCF-7 and MDA-MB-231 cells were seeded in 12 well plates and scratch assay was performed in the presence of TNF-α. **b**, **c** MCF-7 and MDA-MB-231 cells were seeded at low denisty as described in methodology and their ability to form colonies in the presence and absence of TNF-α,2-DG, NAC, and pyruvate was monitored. **d** MDA-MB-231 cells were treated with TNF-α either alone or in combination with Hemin (10 mM) and the cell viability was assessed as described in methodology by MTT

To further investigate the role of TNF-α modulated mitochondrial OXPHOS complex activity in regulating tumorigenic potential of ER/PR +ve: MCF-7 and ER/PR-ve: MDA-MB-231 cells, we analyzed the clonogenic ability in the presence/absence of TNF-α. Interestingly, TNF-α inhibits the clonogenic ability of MCF-7 cells whereas enhances clonogenicity of MDA-MB-231 cells (Fig. 5b). The addition of 2DG in the presence of TNF-α further reduced clonogenic ability of the MDA-MB-231 cells suggesting the glycolysis is essential for MDA-MB-231 cells. The rescue of clonogenicity was observed in the presence of NAC (N-Acetyl cysteine), a ROS scavenger in MDA-MB-231 cells, whereas it was not observed in the MCF-7 cells. Previously, it had been observed that electron acceptors are limited to drive ETC and other anaplerotic reactions in cancer cells (TCA in cancer); hence, we monitored the clonogenic ability in the presence of pyruvate. The presence of pyruvate can rescue clonogenic ability in the presence of TNF-α in MCF-7 (Fig. 5c). Interestingly, there was no major change in pyruvate stimulated clonogenic ability of MDA-MB-231 cells in the presence of TNF-α. We also checked the migration ability of both cells in the presence of TNF-α and observed that TNF-α enhanced the migration ability of MDA-MB-231 cells as compared to MCF-7 (Fig. 5a). This is further supported by the increase in the number of colony-forming units in culture medium supplemented with pyruvate. This suggests that it is not ROS but electron acceptor ability that maintains the ratio of NAD/NADH in the cell to drive the TCA cycle and glycolysis in MCF-7, whereas pyruvate, the electron acceptor, is not the limiting factor [28, 29].

Hemin is known to degrade BTB and CNC homology1 (BACH1), a haem binding transcription factor that is increased in TNBC tumors and enhance mitochondrial respiratory activity[30]. To further confirm the reliance of MDA-MB-231 on OXPHOS activity, we checked for cell viability of MDA-MB-231 in the presence of TNF-α in combination with Hemin. We observed that the cell viability of MDA-MB-231 cells significantly decreased in the presence of TNF-α and Hemin (Fig. 5d). This suggests that enhancing the mitochondrial proteins and functions in MDA-MB-231 cells in the presence of TNF-α can inhibit the triple-negative breast cancer cell survival.

### Subunit of mitochondrial complexes negatively correlates with survival of the breast cancer patients

**Fig. 6:**
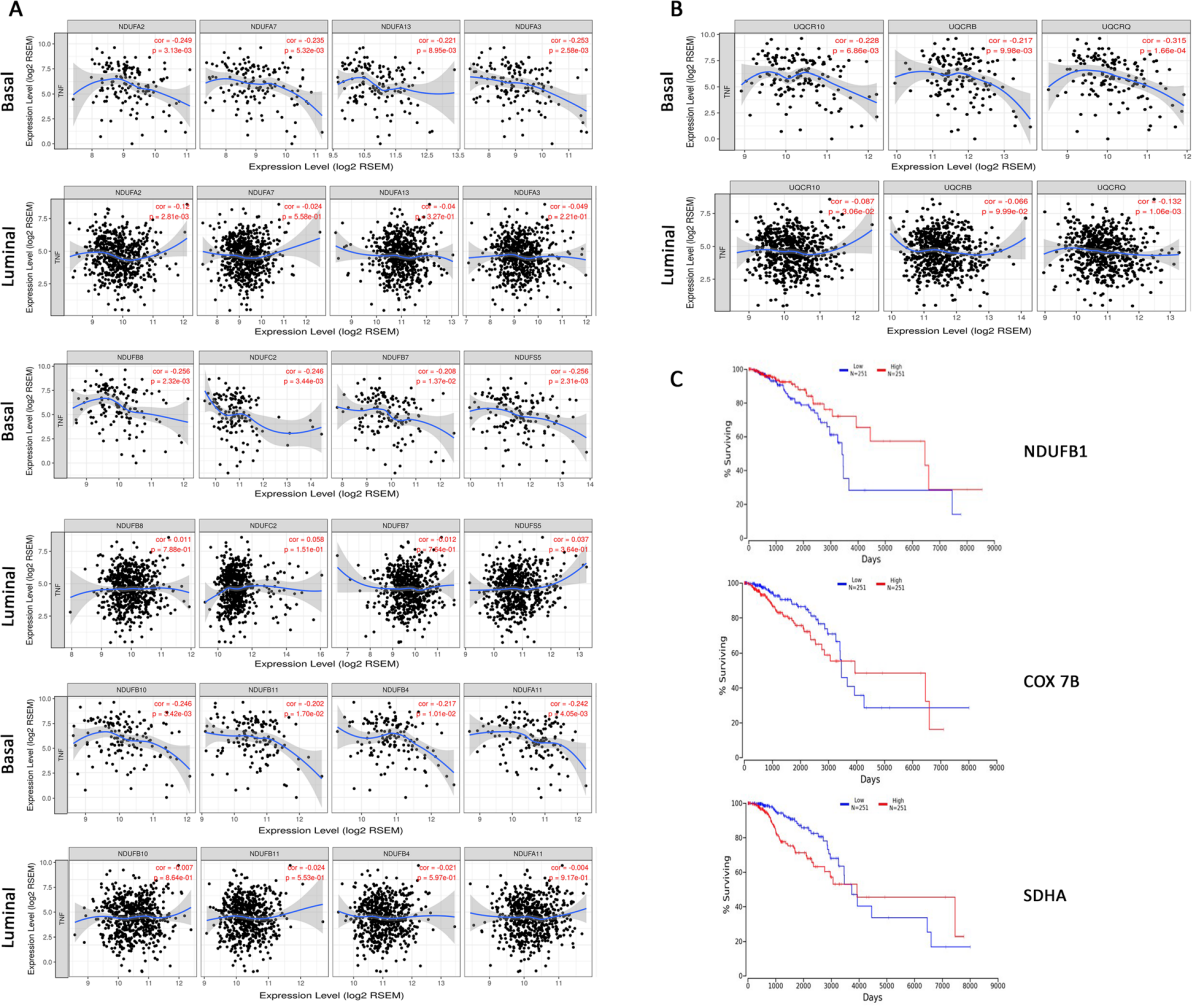
Subunit of complex negatively correlates with survival of the breast cancer patients. **a**, **b** Gene expression analysis between TNF-α and mitochondrial CI and CIII proteins in Basal and Luminal breast cancer patients using TIMER database, respectively. **c** Kaplan-Meier survival plot of BRCA patients in low/high expression of mitochondrial NDUFB1, COX7B, and SDHA genes

The TIMER database is a web resource used for systemic analysis and evaluation of clinical impacts of different immune factors in diverse cancer types hence we analyzed the correlation between TNF-α and the expression level of identified DEPs. The TIMER data showed that increased expression of TNF-α in basal breast cancer patients is associated with decreased gene expression of subunits of mitochondrial complex I. This negative correlation between TNF-α and complex I subunits is significantly higher in basal breast cancer patients (Fig. 6a) and no significant correlation in luminal breast cancer patients was observed. CIII subunits like UQCR10, UQCRB, and UQCRQ expression were also altered in basal breast cancer patients which showed a significant negative correlation with TNF-α expression as compared to luminal breasts cancer patients (Fig. 6b).

We also checked the survivability of breast cancer patients using the Kaplan-Meier survival plot analysis. The high expression of subunits of mitochondrial complexes like NDUFB1 (*p* = 0.052), SDHA (*p* = 0.011), and COX7B (*p* = 0.064) showed an increased percentage of survival and an increased number of survival days (Fig. 6c).

These evidence strongly suggest that TNF-α differentially regulates the mitochondrial subunits in luminal and basal breast cancer patients and determine the survival rate and span of the breast cancer patients.

### Discussion

Increased level of cytokines in TME of solid tumors may modulate mitochondrial function for metabolic adaptation; however, its systemic role in the regulation of mitochondrial proteome and OXPHOS assembly is not well understood. The regulation of TNF-α mediated mitochondrial complex assembly and its role in the regulation of tumorigenic potential of different breast cancer cell types had not been systemically investigated. To understand the differential regulation, we here systemically characterized mitochondrial proteome of two different cell types MCF-7: ER/PR +ve, responsive representing early tumor conditions, and MDA-MB-231 cells: ER/PR −ve representing aggressive and metastatic conditions tumor cell types. There is no systemic study monitoring the mitochondrial proteome in different breast cancer cells which determines differential metabolic adaptations in TME. There are some previous reports where TRAIL, a member of the TNF-α family, modulated total cellular proteome had been analyzed [31]; however, total cellular proteome truly does not reflect mitochondrial proteins [32]. Hence, it is important to understand the TNF-α modulated mitochondrial proteome to understand the differential mitochondrial role in driving the tumor characteristic and heterogeneity.

The high-resolution proteomics clearly showed that TNF-α differentially modulates mitochondrial proteome of MDA-MB-231 and MCF-7 cells leading to differential mitochondrial function and OXPHOS capacity. Other pathways that are differentially regulated in the presence of TNF-α like sirtuin pathways and iron homeostasis in MCF-7 and MDA-MB-231 cells also regulate mitochondrial functions; however, this can be a topic of further investigation. The study further focused on the assembly of mitochondrial respiratory chain complexes which are differentially regulated in MCF-7 and MDA-MB-231 cells. The level of mitochondrial DNA encoded transcripts specifically ND2 and ND3 which form part of the core unit of the complex I, increased in MCF-7, whereas remaining unaltered in MDA-MB-231 cells. The levels of NDUFS3 ( N module component) and NDUFB1 (the component of ND4 module) were significantly decreased in MCF-7 cells in the presence of TNF-α but, however, increased in MDA-MB-231 suggesting that TNF-α can differentially modulate the complex I activity which is in consonance with in-gel activity and super complex assembly.

Similarly, mitochondrial complex III, which can accept electrons from both complex I and complex II through ubiquinone is critical for mitochondrial function. The study here showed a decrease in complex III activity which correlates with mitochondrial proteomics data. The level of assembly factor LYRM7 decreased in MDA-MB-231 cells compared to MCF-7 cells. Emerging reports suggest that binding of HSC20 (co-chaperone) to the LYR motif of LYRM7 in a pre-assembled UQCRFS1-LYRM7 intermediate in the mitochondrial matrix facilitates Fe-S cluster transfer to UQCRFS1, hence assembly of complex III (Chaperon CI-III). This decrease in LYRM7 in mitochondria of MDA-MB-231 cells strongly suggests that incorporation of Fe-S complexes in mitochondrial electron transport chain may be modulated in mitochondrial complex III. Hence, TNF-α modulates flux of NADH which will shift the TCA cycle intermediates towards anaplerotic reactions in aggressive breast tumor cells (MDA-MB-231). This is further supported by the analysis of clonogenic abilities of the cells in the presence of TNF-α. Interestingly, we observed that TNF-α inhibited the clonogenic ability of MCF-7 cells which is rescued in the presence of pyruvate suggesting that electron acceptors are limiting factor. Pyruvate level can be differentially regulated in MCF-7/MDA-MB-231 cells, which is known to act as an electron acceptor and can determine the cell proliferation. Interestingly, in MDA-MB-231, highly metastatic cells, pyruvate is not a limiting factor as TNF-α reprogrammed the activity of OXPHOS for anaplerotic reaction, as we observed pyruvate supplementation showed no major effect on cell proliferation or clonogenic abilities of the cells. This observation further supported as we observed a high level of pyruvate in MDA-MB-231 cells as compared to MCF-7 cells (unpublished observation).

The decrease in complex I/III activity may increase the level of ROS in triple-negative aggressive breast cancer cells which may act as mitohormetic response rather than cell death. This observation is in consonance with a recent report where it had been observed that an increased level of ROS in selected aggressive breast cancer cells from TNBC patients can induce mitohormetic response in modulating nuclear genes which help to survive in the hostile tumor microenvironment.

The overall decrease in the mitochondrial proteins and complex activity in MDA-MB-231 is supported by a recent study where BTB and CNC homology1 (BACH1), a haem binding transcription factor increased in tumors from patients with TNBC which negatively regulates transcription of electron transport chain (ETC) genes [30]. It was observed that enhancing the reliance of breast cancer cells to mitochondrial functions by modulating the transcription factor BACH1 using hemin which initiates degradation of BACH1, sensitizes the cancer cells to metformin. In our study, the TIMER webserver showed a negative correlation of CI and CIII subunits with TNF-α in basal breast cancer patients. This correlates with the decrease in complex assembly and activity in MDA-MB-231 in the presence of TNF-α. Further, the survivability of breast cancer patients also correlates with the expression of mitochondrial complexes.

## Conclusion

In conclusion, the subcellular proteomics identifies the differential behavior of the ER/PR +ve and ER/PR −ve breast cancer cells in response to TNF-α. The evidences here clearly suggest that TNF-α modulates metabolism differentially in ER/PR +ve and ER/PR −ve breast cancer cells by modulating the levels of critical assembly factors and subunits involved in mitochondrial respiratory chain supercomplexes. TNF-α modulated metabolic reprogramming favors survival and proliferation of more aggressive ER/PR −ve breast cancer cells. This study identified novel assembly factors as possible therapeutic target to prevent the progression of aggressive breast cancer cells hence the survival of the breast cancer patients.

## Supplementary Information

**Additional file 1: Figure S1.** Proteomic profiling of mitochondrial proteins in MCF-7 and MDA-MB-231 under TNF-α stimulation. (A) Heat map of a hierarchical clustering showing the expression patterns of proteins of the mitochondria. The fold change scale represents a sample of the mean-subtracted average of the regularized log-transformed read counts in each sample. The up-regulated proteins are in red and down-regulated proteins are in green(B) Cellular processes of DEPs by IPA tool. **Figure S2.** Biological network analysis of DEPs. Associations among DEPs are shown by gray lines, which represent direct or indirect interactions. Upregulated proteins are shown in red, and downregulated proteins are shown in green. (A)& (B) DEPs in MCF-7 and MDA-MB-231 in presence of TNF-α respectively.

## Data Availability

The datasets used and/or analyzed during the current study are available from the corresponding author on reasonable request.

## Abbreviations

TME: Tumor microenvironment
TNF-α: Tumor necrosis factor alpha
ER: Estrogen receptor
PR: Progesterone receptor
TAM: Tumor-associated macrophages
OXPHOS: Oxidative phosphorylation
ETC: Electron transport chain
TIMER: Tumor Immune Estimation Resource
ATP: Adenosine triphosphate
ROS: Reactive oxygen species
NAC: N-acetylcysteine
TNBC: Triple-negative breast cancer
