# Supplementary material for: TNF-α differentially modulates subunit levels of respiratory electron transport complexes of ER/PR +ve/−ve breast cancer cells to regulate mitochondrial complex activity and tumorigenic potential
Source: Cancer Metab. 2021 Apr 29;9:19. doi: 10.1186/s40170-021-00254-9 (PMC8082668; doi:10.1186/s40170-021-00254-9)
Supplement: Supplementary file 1 — Additional file 1: Figure S1. Proteomic profiling of mitochondrial proteins in MCF-7 and MDA-MB-231 under TNF-α stimulation. (A) Heat map of a hierarchical clustering showing the expression patterns of proteins of the mitochondria. The fold change scale represents a sample of the mean-subtracted average of the regularized log-transformed read counts in each sample. The up-regulated proteins are in red and down-regulated proteins are in green(B) Cellular processes of DEPs by IPA tool. Figure S2. Biological network analysis of DEPs. Associations among DEPs are shown by gray lines, which represent direct or indirect interactions. Upregulated proteins are shown in red, and downregulated proteins are shown in green. (A)& (B) DEPs in MCF-7 and MDA-MB-231 in presence of TNF-α respectively. [file 40170_2021_254_MOESM1_ESM.docx]

**Supplementary figure:**


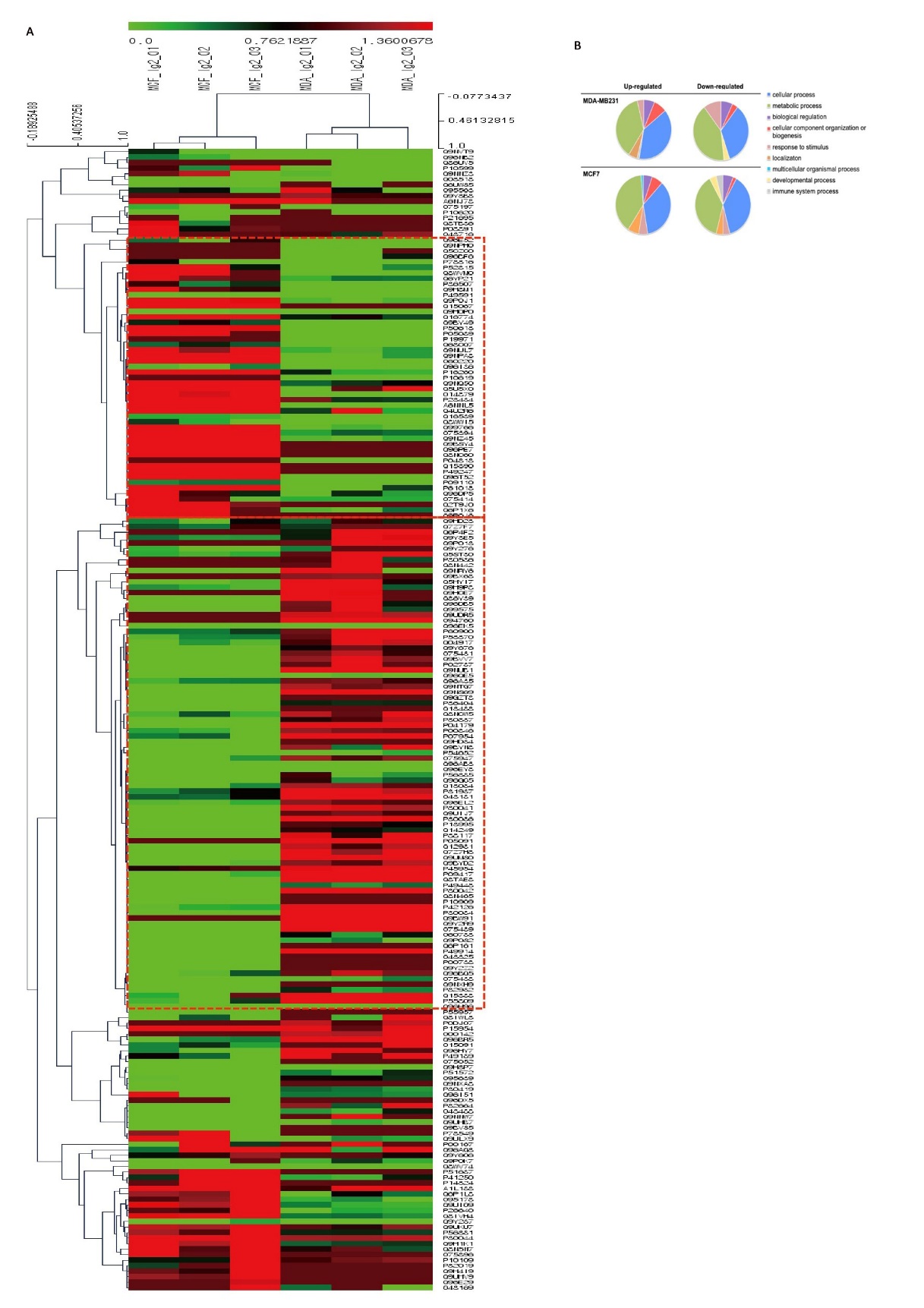


***Figure S1:Proteomic profiling of mitochondrial proteins in MCF-7 and MDA-MB-231 under TNF-α stimulation.***

***(A)****Heat map of a hierarchical clustering showing the expression patterns of proteins of the mitochondria. The fold change scale represents a sample of the mean-subtracted average of the regularized log-transformed read counts in each sample. The up-regulated proteins are in red and down-regulated proteins are in green****(B)****Cellular processes of DEPs by IPA tool****.***


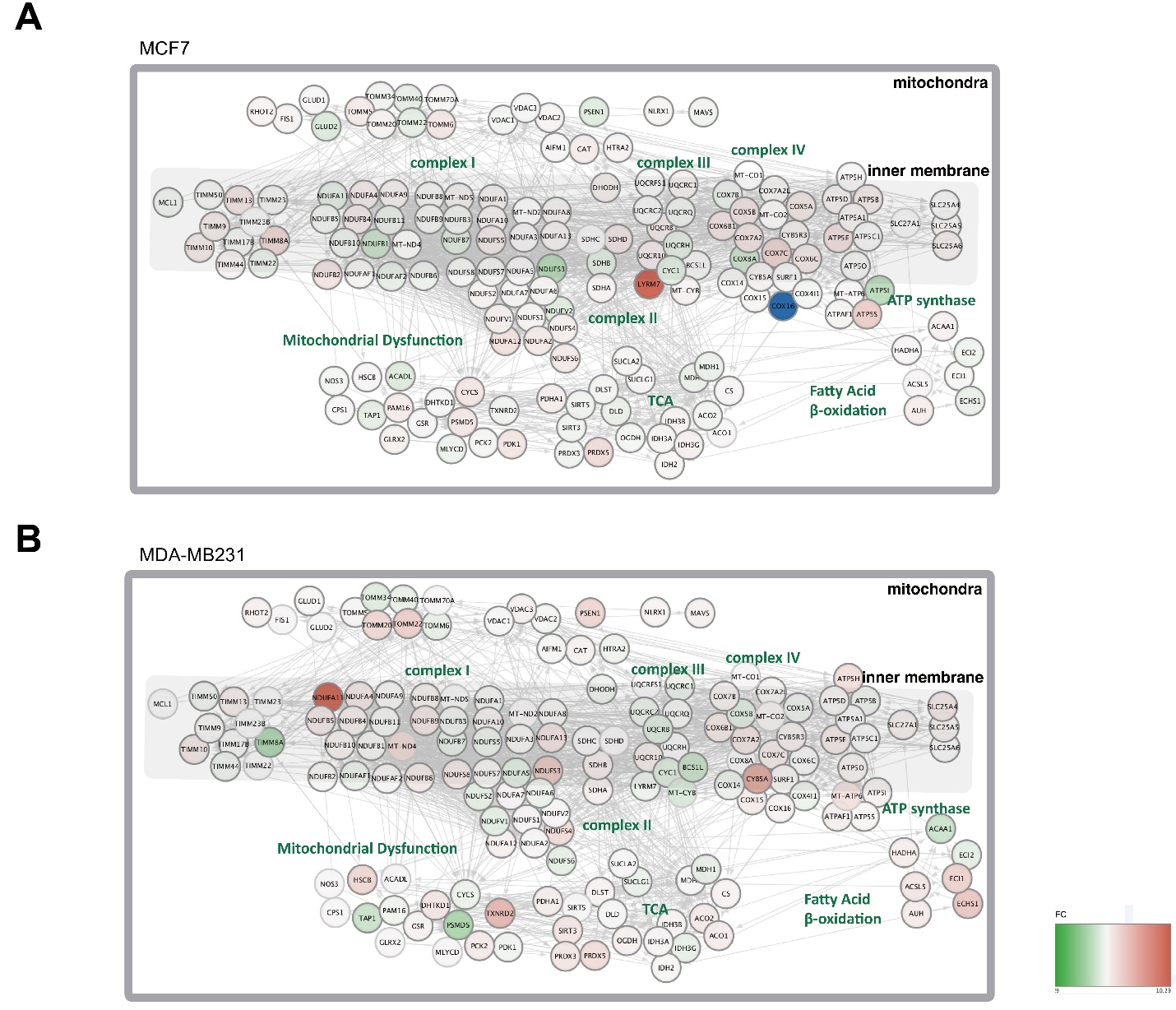


***Figure S2:Biological network analysis of DEPs.*** *Associations among DEPs are shown by gray lines, which represent direct or indirect interactions. Upregulated proteins are shown in red, and downregulated proteins are shown in green****. (A)& (B)*** *DEPs in MCF-7 and MDA-MB-231 in presence of TNF-α respectively*
